# Promoting functions of microRNA-29a/199B in neurological recovery in rats with spinal cord injury through inhibition of the RGMA/STAT3 axis

**DOI:** 10.1186/s13018-020-01956-4

**Published:** 2020-09-18

**Authors:** Weijie Yang, Ping Sun

**Affiliations:** Department of Orthopedics, Shanghai Eighth People’s Hospital, No.8, Caobao Road, Shanghai, 200235 People’s Republic of China

**Keywords:** MicroRNA-29a; MicroRNA-199B, RGMA, Spinal cord injury, STAT3 signaling pathway; Neurological recovery

## Abstract

**Background:**

The prognostic and therapeutic potential of microRNAs (miRNAs) in spinal cord injury (SCI) has aroused increasing concerns. This study aims to research the functions of miR-29a/199B in the neurological function recovery after SCI and the mechanical mechanism.

**Methods:**

A rat model with SCI was induced with sham-operated ones as control. The locomotor function and coordination of rat hindlimbs were determined by a Basso, Beattie, and Bresnahan (BBB) locomotor rating scale and a ladder-climbing test, respectively. Expression of a neurofilament protein NF-200 and synaptophysin in gray matter of rats was determined to evaluate neuronal recovery in a cellular perspective. Binding relationships between miR-29a/199B with RGMA were predicted and validated using luciferase assays. Altered expression of miR-29a/199B and RGMA was introduced to explore their functions in rat neurological functions. The protein level and phosphorylation of STAT3 in gray matter were measured by western blot analysis.

**Results:**

miR-29a and miR-199B were poorly expressed, while RGMA was abundantly expressed in gray matter at injury sites. Either miR-29a or miR-199B could bind to RGMA. Overexpression of miR-29a/199B or silencing of RGMA led to an increase in BBB locomotor scores, hindlimb coordination ability, and the expression of NF-200 and synaptophysin in gray matter. Further inhibition in miR-29a/199B blocked the promoting roles of RGMA silencing in neurological recovery. Upregulation of miR-29a/199B or downregulation of RGMA suppressed the phosphorylation of STAT3.

**Conclusion:**

This study evidenced that miR-29a and miR-199B negatively regulated RGMA to suppress STAT3 phosphorylation, therefore promoting the neurological function recovery in rats following SCI.

## Background

Traumatic spinal cord injury (SCI) is usually caused by an external physical impact damage to the spinal cord and contributes to everlasting neurological impairment and dysfunction and burdens individuals, families, and the society with a high economic cost [1]. Even though survive the initial injury, the SCI patients face great risks of medical complications throughout their lives, which is often related to infectious morbidity, leading to significant reductions in life quality and expectancy [2]. Advanced therapies such as decompression, functional stimulation, neurorehabilitation, nerve and tendon transfers, and neural bypass, as well as drug treatments, surgery, and cell transplantation, are currently promising strategies for SCI, but there are no available regenerative treatments to date to cure the disease [3, 4]. Understanding of molecular biomarkers may help program candidacy and tailor treatment for patients [4], and therefore, identifying novel molecular biomarkers and mechanisms holds great potential to improve the outcome of SCI patients.

MicroRNAs (miRNAs) are reported as one of the important biomarkers associated with the injury severity and prognosis of SCI, which may predict spontaneous neurological recovery over time with high precision, sensitivity, and reproducibility [5]. Thanks to their unique potent function in regulating target gene expression post-transcriptionally, miRNAs are emerging potential tools or targets for SCI treatment with great therapeutic significances [6, 7]. miR-29a and miR-199B have been noted to promote axonal regeneration and neuronal functional recovery or reduce the inflammatory response in SCI [8, 9], but their functions and implicated mechanisms in SCI progression are not fully studied yet. Interestingly, the online prediction on a bioinformatics system StarBase (http://starbase.sysu.edu.cn/) in our study predicted that these two miRNAs shared a binding relationship with the same target gene, repulsive guidance molecule A (RGMA). RGMA is a GPI-linked glycoprotein that exists in soluble and membrane-bound forms and suppresses neurite growth by binding to neogenin, its neuronal receptor [10]. Correspondingly, inhibition of either RGMA or neogenin was found to promote neuronal survival and functional recovery after SCI [11, 12]. The signal transducer and activator of transcription 3 (STAT3) signaling is implicated in multiple inflammatory responses by regulating some pro-inflammatory cytokines such as interleukin-6 (IL-6) and IL-17, and its downregulation was found to reduce spinal cord neuroinflammation and promote neuronal functional recovery after SCI [13, 14]. In addition, STAT3 is expressed by neurons, astrocytes, and other cell types in the central nervous system (CNS), and it is significantly activated following CNS insults [15]. Taken the above discussion together, we speculated that miR-29a/miR-199B possibly targets RGMA to relieve SCI. Therefore, a rat model with SCI was established [16, 17] to validate this hypothesis and to explore whether STAT3 is involved in the events.

## Materials and methods

### Ethics statement

The study was performed with the approval of the Ethical Committee of Shanghai Eighth People's Hospital (Approval No. 2019011). All experimental procedures were conducted in line with the ethical guidelines for the study of experimental pain in conscious animals. Great efforts were made to minimize the usage and suffering of animals.

### Establishment of a rat model with SCI

A total of 80 male Sprague-Dawley rats (8 weeks old, 200–220 g) purchased from the Experimental Animal Center of Sun Yat-sen University were housed in specific-pathogen-free (SPF) grade animal rooms with controlled humidity (60–65%) and temperature (22–25 °C). The mice were allowed ad libitum feed and water and acclimated in a 12-h-dark/light cycle for 1 week. Thereafter, 72 rats were used for SCI model establishment. In brief, the rats were inhaled with 2% isoflurane and the mixture of Nitrous oxide and oxygen (1:2, v/v) for anesthesia. Under a sterile condition, the T9-T11 laminae were removed by laminectomy, and then the T10 segment was compressed using a modified aneurysm clip at 20 g for 60 s. Pain was alleviated by analgesics, while the infection was prevented with antibiotics. Successful model establishment in rats was confirmed by a < 1 score 1 h after surgery according to a Basso, Beattie, and Bresnahan (BBB) locomotor rating scale [18, 19]. The remaining 8 rats were subjected to sham operation with only the skin and muscle cut open and sutured.

After model establishment, each model rat was injected with 2.5 μL lentiviral vector (LV) at 3 mm next to the lesion site. The needle was maintained for 5 min and quickly pulled out to avoid backflow. The injection was performed twice a week for a total of 6 weeks. Thereafter, the animals were euthanized through intraperitoneal injection of 150 mg/kg pentobarbital sodium and then the tissues from the gray matter in the lesion sites. The animals were allocated into the following groups after the corresponding injection: SCI group (model group without transfection), negative control (NC) mimic group (7.5 × 10^5^ TU LV-NC mimic), miR-29a mimic (7.5 × 10^5^ TU LV-miR-29a mimic), miR-199B mimic (7.5 × 10^5^ TU LV-miR-199B mimic), short hairpin (sh)-NC group (7.5 × 10^5^ TU LV-sh-NC), sh-RGMA (7.5 × 10^5^ TU LV-sh-RGMA), sh-RGMA + NC inhibitor group (7.5 × 10^5^ TU LV-sh-RGMA + NC inhibitor), sh-RGMA + miR-29a inhibitor (7.5 × 10^5^ TU LV-sh-RGMA + miR-29a inhibitor), and sh-RGMA + miR-199B inhibitor (7.5 × 10^5^ TU LV-sh-RGMA + miR-199B inhibitor) (*n* = 8 in each group).

### Dual-luciferase reporter gene assay

The binding relationships between miR-29a/199B and RGMA were predicted on StarBase. Then, the putative wild-type (WT) RGMA 3′UTR fragment was designed and inserted into pMIR-reporter plasmids (Promega, Madison, WI, USA) to construct pMIR-RGMA-WT vectors, and the corresponding mutant-type (MUT) binding sites between RGMA 3′UTR and miR-29a/199B were used to construct pMIR-RGMA-MUT vectors. Well-constructed WT and MUT vectors were co-transfected with miR-29a/199B mimic or NC mimic into HEK293T cells (Beinuo Biotechnology Co., Ltd., Shanghai, China). Forty-eight hours later, the relative luciferase activity was measured on a Dual-Luciferase Reporter Assay System (Promega).

### Immunofluorescence staining

The longitudinal sections of the spinal tissues were collected from rats on the 6th week. The sections were fixed in 40 g/L paraformaldehyde for 15 min, washed in phosphate buffer saline (PBS), and blocked in PBS containing 0.1% Triton X-100 and 5% bovine serum albumin (BSA) at 37 °C for 50 min. Next, the slides were incubated with neurofilament 200 antibody (NF-200, 1:200, #55453, Cell Signaling Technology (CST), Beverly, MA, USA) at 4 °C overnight and then with cy3-labeled secondary antibody goat anti-rabbit immunoglobulin G (IgG) (ab6939, 1:500, Abcam Inc., Cambridge, MA, USA) at room temperature for 60 min. Then, the slides were sealed and observed under a LSM510 meta confocal microscope (Carl Zeiss MicroImaging, Inc., Thornwood, NY, USA). The gray matter at the region 2–3 mm posterior to the spinal injury site was used for measurement. Relative NF-200-positive area was calculated using an Image J 2.0 software.

### BBB locomotor rating scores

The hindlimb motor function of rats was evaluated using the BBB locomotor rating scale on 1, 2, 3, 4, 5, and 6 weeks after model establishment, respectively. Briefly, the rats were placed in an open box and stimulated to move forward. The hip, knee, ankle flexibility, and the movement and coordination were evaluated. If the rats had isolated joint movements with no or little hindlimb movement, the rat would be allocated into an early stage (score 0–7). Rats had occasional uncoordinated stepping would be classified into an intermediate stage (score 8–13). Rats that were capable of hindlimb coordination, equilibrium, and stepping were categorized into a late stage (score 14–21) [20].

### Ladder-climbing test

Locomotion of animals was further determined using a ladder-climbing test. On 1, 2, 3, 4, 5, and 6 weeks, each group of rats was placed on a 1-m tall ladder with uneven intervals. The number of times of rat slips (error steps) was counted and scored with a higher score indicating worse coordination. The total steps of hindlimbs of each rat were recorded as well by two observers. Three independent experiments were performed and the average value was evaluated. The success rate was calculated as follows: success rate = 1 − slip steps/total steps × 100%.

### Western blot analysis

Tissues from the gray matter at the spinal lesion sites were collected and lysed in radio-immunoprecipitation assay lysis buffer (Boster Biological Technology Co., Ltd., Wuhan, Hubei, China), and the protein concentration was determined using a bicinchoninic acid kit (70-PQ0012, MultiSciences, Zhejiang, China). Then, the protein was separated on sodium dodecyl sulfate-polyacrylamide gel electrophoresis and transferred to polyvinylidene fluoride membranes. The membranes were blocked with 5% BSA for 1 h and then incubated with the primary antibodies to RGMA (1:10000, ab169761, Abcam), NF-200 (1:1000, #55453, CST), synaptophysin (1:1000, ab14692, Abcam), STAT3 (1:1000, ab68153, Abcam), p-STAT3 (1:1000, ab32143, Abcam), and glyceraldehyde-3-phosphate dehydrogenase (GAPDH, 1:2000, ab9485, Abcam) at 4 °C overnight. Next, the membranes were incubated with the horseradish peroxidase (HRP)-labeled goat anti-mouse IgG (1:2000, ab205719, Abcam) at room temperature for 1 h. The western blots were developed using an enhanced chemiluminescence kit (BB-3501, Amersham Pharmacia Biotech, Piscataway, NJ, USA) exposed by a Gel imager, captured by a Bio-Rad image analysis system (Bio-Rad, Inc., Hercules, CA, USA), and analyzed using the Quantity One v4.6.2 software.

### Reverse transcription-quantitative polymerase chain reaction (RT-qPCR)

Total RNA from tissues was collected using a TRIzol kit (15596026, Invitrogen, Inc., Carlsbad, CA, USA) and reversely transcribed into cDNA using a PrimeScript RT reagent Kit (RR047A, Takara, Holdings Inc., Kyoto, Japan). The cDNA was amplified for qPCR using a Fast SYBR Green PCR Kit (Applied Biosystems, Inc., Carlsbad, CA, USA) on an ABI PRISM 7300 System (Applied biosystems). The primers are shown in Table 1 with U6 as an internal reference for miR-29a and miR-199B while GAPDH for mRNAs. Relative gene expression was determined by the 2^-△△Ct^ method.

**Table 1:** Primer sequences for RT-qPCR

Gene	Primer sequence (5′-3′)
miR-29a	F: GCGCACTGATTTCTTTTGGTGTTCAG
	R: GCGAGCACAGAATTAATACGAC
miR-199B	F: TTATCCTAATTGCTCCTACGGCT
	R: ATTCGGCATCGCGCTAAACGTTA
RGMA	F: TCACCGACCGCTTCCAGACC
	R: CTCCTTCACCAGTTACGACACCTC
GAPDH	F: GGGAGCCAAAAGGGTCAT
	R: GAGTCCTTCCACGATACCAA
U6	F: CGAACGATACAGAGAAGATTAGC
	R: CGAACGATACAGAGAAGATTAGC

*RT-qPCR* reverse transcription-quantitative polymerase chain reaction, *miR* microRNA, *RGMA* repulsive guidance molecule A, *GAPDH* glyceraldehyde-3-phosphate dehydrogenase, *F* forward, *R* reverse

### Statistical analysis

SPSS 21.0 (IBM Corp. Armonk, NY, USA) was used for data analysis. Measurement data were expressed as mean ± standard deviation (SD) from at least three independent experiments. Differences between two groups were analyzed by the *t* test, while data among multiple groups were compared by one-way or two-way analysis of variance (ANOVA), followed by Tukey’s multiple comparison test.

## Results

### miR-29a and miR-199B were downregulated in rats with SCI

**Fig. 1:**
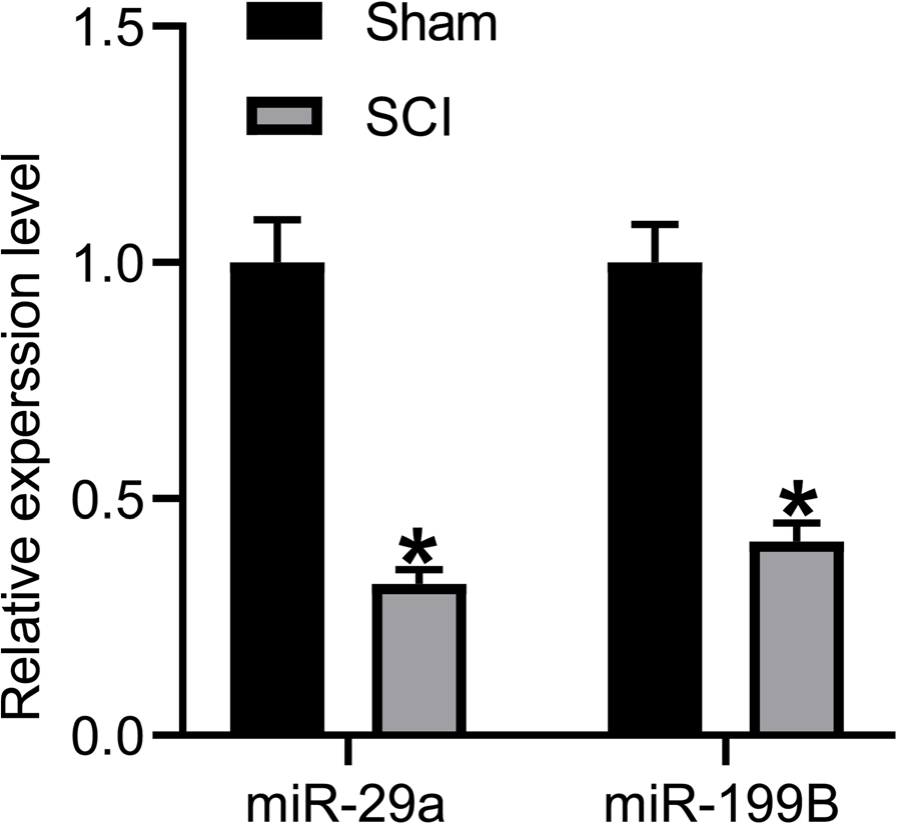
miR-29a and miR-199B are downregulated in rats with SPI. miR-29a and miR-199B expression in the gray matter in rats determined by RT-qPCR. *N* = 8. Data were presented as mean ± SD from at least three independent experiments. Data were compared by two-way ANOVA. *p < 0.05

As mentioned before, miR-29a and miR-199B were reported to be poorly expressed in rat models with SCI and showed protective roles in neuronal recovery. But the detailed functions of these two molecules and the involving mechanisms in SCI remain largely unknown. Here, we established a rat model with SCI. The successful model establishment in rats was confirmed by < 1 BBB rating score 1 h after surgery. According to the RT-qPCR results, miR-29a and miR-199B were poorly expressed in the gray matter in rats with SCI compared to the sham-operated ones (*p* < 0.05) (Fig. 1).

### Overexpression of miR-29a or miR-199B reduces neuronal injury and promotes the locomotor function of SCI rats

**Fig. 2:**
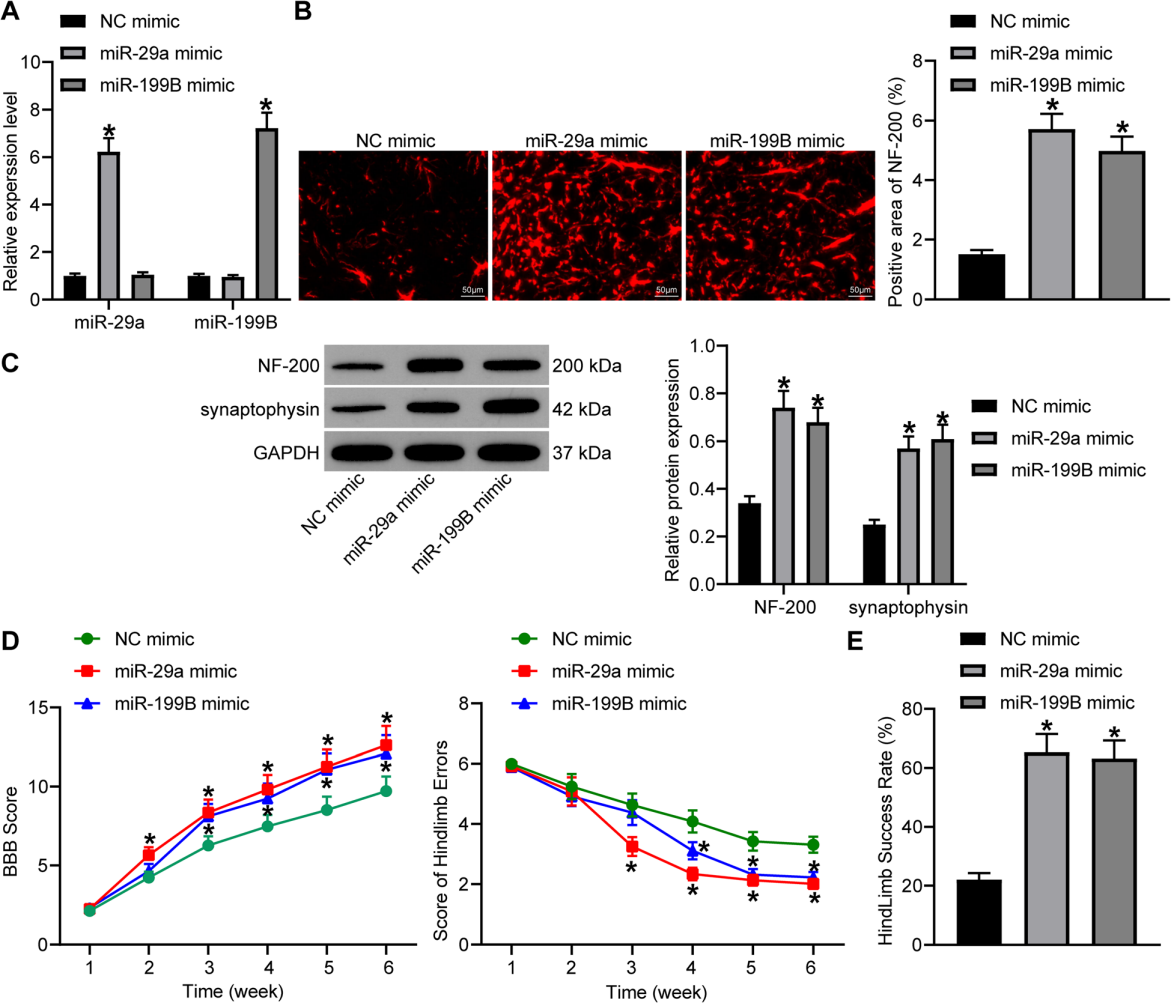
Overexpression of miR-29a or miR-199B promotes neurological function recovery in rats with SCI. **a** miR-29a and miR-199B expression in the gray matter in the injury sites determined by RT-qPCR. **b** NF-200 expression in the gray matter 2–3 mm posterior to the injury sites using immunofluorescence staining. **c** Protein levels of NF-200 and synaptophysin in the gray matter 2–3 mm posterior to the injury sites detected by western blot analysis. **d** The weekly locomotor function of rats evaluated using the BBB rating scale. **e** Coordination of rat hindlimbs measured by a ladder-climbing test. *N* = 8. Data were presented as mean ± SD from at least three independent experiments. Data were compared by one-way or two-way ANOVA. *p < 0.05

To explore the functions of miR-29a and miR-199B, miR-29a or miR-199B was overexpressed in model rats through delivering miR-29a/199B mimic. The successful transfection was validated according to the RT-qPCR results (Fig. 2a). Compared to NC mimic, transfection of miR-29a mimic or miR-199B mimic led to a significant increase in miR-29a/199B expression (all *p* < 0.05).

We then measured the expression of a neurofilament protein NF-200 in the gray matter 2–3 mm posterior to the injury sites using immunofluorescence staining. It was found that the staining intensity of NF-200 was increased upon miR-29a or miR-199B overexpression (all *p* < 0.05) (Fig. 2b). In addition, the western blot analysis that the protein levels of NF-200 and synaptophysin were increased after miR-29a and miR-199B upregulation (Fig. 2c).

The neurologic function of rats was monitored weekly using a BBB locomotor rating scale (Fig. 2d). The BBB locomotor scores of rats were significantly increased on the 2nd and 3rd week, respectively, after miR-29a mimic or miR-199B mimic administration (all *p* < 0.05). A ladder-climbing test was used to evaluate the coordination of rat hindlimbs. It was found the time of slips was notably decreased, and correspondingly, the success step rate was notably increased since the 3rd week after miR-29a mimic transfection. Likewise, the error score was reduced while success rate was increased from the 4th week in rats with miR-199B overexpression (all *p* < 0.05) (Fig. 2e). Collectively, these results suggested that overexpression of miR-29a or miR-199B promotes neurological function recovery in rats with SCI.

### miR-29a and miR-199B target RGMA and suppress the STAT3 signaling pathway

**Fig. 3:**
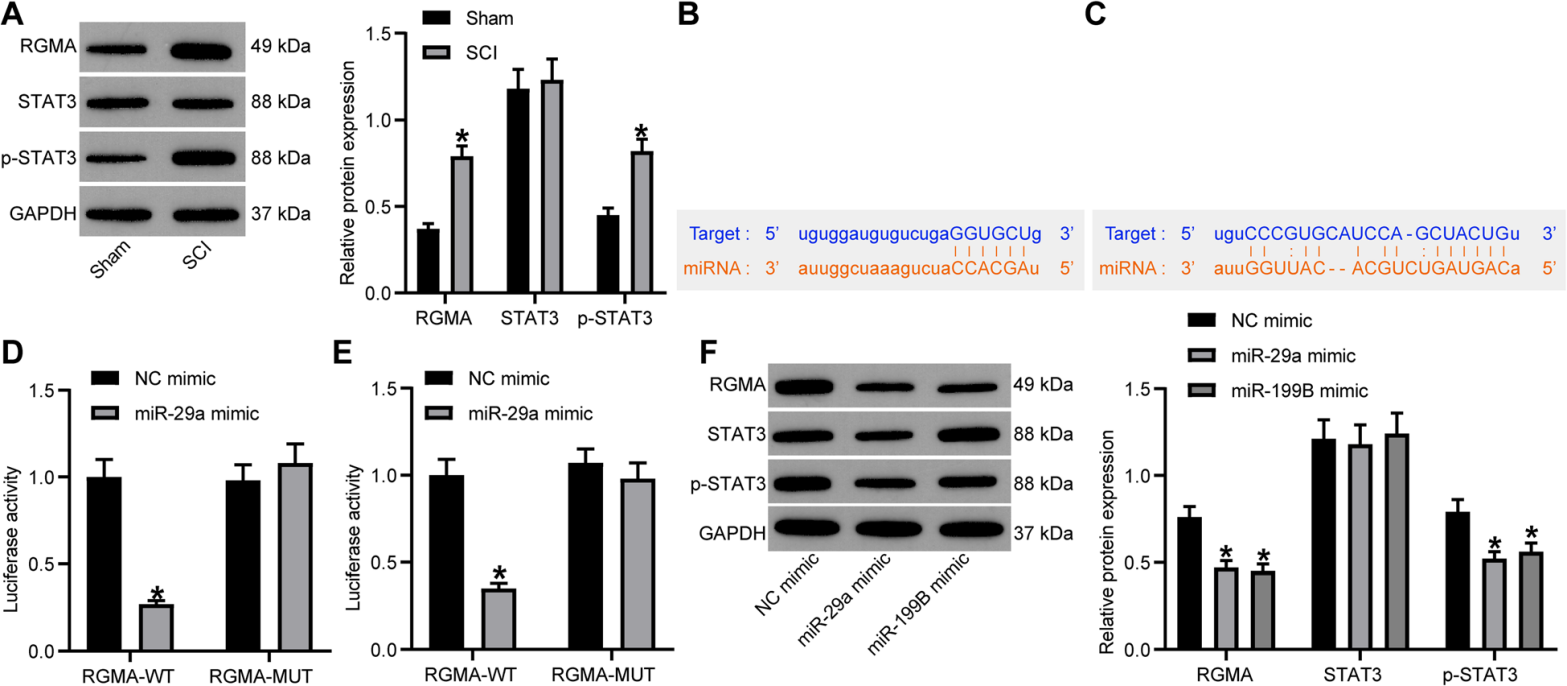
miR-29a and miR-199B negatively regulate RGMA and inactivate the STAT3 signaling pathway. **a** Protein levels of RGMA and STAT3 as well as phosphorylation of STAT3 in tissues in gray matter of SCI model rats determined by western blot analysis. **b**, **c** Putative binding sites between miR-29a (**b**) and miR-199B (**c**) and RGMA predicted on StarBase (http://starbase.sysu.edu.cn/). **d**, **e** Binding relationships between miR-29a (**d**) and miR-199B (**e**) and RGMA validated through luciferase assays. **f** Protein levels of RGMA and STAT3 as well as phosphorylation of STAT3 in tissues in gray matter of SCI model rats after miR-29a/199B transfection determined by western blot analysis. *N* = 8. Data were presented as mean ± SD from at least three independent experiments. Data were compared by one-way or two-way ANOVA. *p < 0.05 vs sham group or NC mimic

RGMA has been noted to be upregulated in SCI rats and suppressed neurological recovery [10], and STAT3 signaling activation was suggested to inhibit nerve regeneration and repair [21]. Interestingly, either miR-29a or miR-199B was reported to negatively regulate the STAT3 signaling pathway [22, 23]. Therefore, we determined the protein levels of RGMA and STAT3 as well as the phosphorylation of STAT3 in the tissues in gray matter. The results showed that the protein level of RGMA and the phosphorylation of STAT3 were notably increased in the SCI model rats relative to the sham-operated ones (all *p* < 0.05), while the total protein level of STAT3 showed little differences between the two groups (*p* > 0.05) (Fig. 3a).

According to the data the bioinformatics system StarBase, we found that either miR-29a or miR-199B had binding sites with RGMA (Fig. 3b, c). Then, the binding relationships between miR-29a/199B and RGMA were further validated through luciferase assays. It was found that co-transfection of miR-29a/199B mimic and pMIR-RGMA-WT vector led to a notable decline in luciferase activity in HEK293T cells (all *p* < 0.05), while the cells transfected with NC mimic or pMIR-RGMA-MUT vector showed little changes in luciferase activity (all *p* > 0.05) (Fig. 3d, e). These results indicated that both miR-29a and miR-199B can directly bind to RGMA.

Thereafter, we explored the correlations between the miR-29a/199B expression and the levels of RGMA and STAT3 in the tissues in gray matter. It was found that the protein level of RGMA and phosphorylation of STAT3 were notably decreased after miR-29a mimic and miR-199B mimic transfection in SCI model rats (all *p* < 0.05), but the STAT3 level showed no significant changes (*p* > 0.05) (Fig. 3f).

### Silencing of RGMA promotes the neurological recovery in rats following SCI

**Fig. 4:**
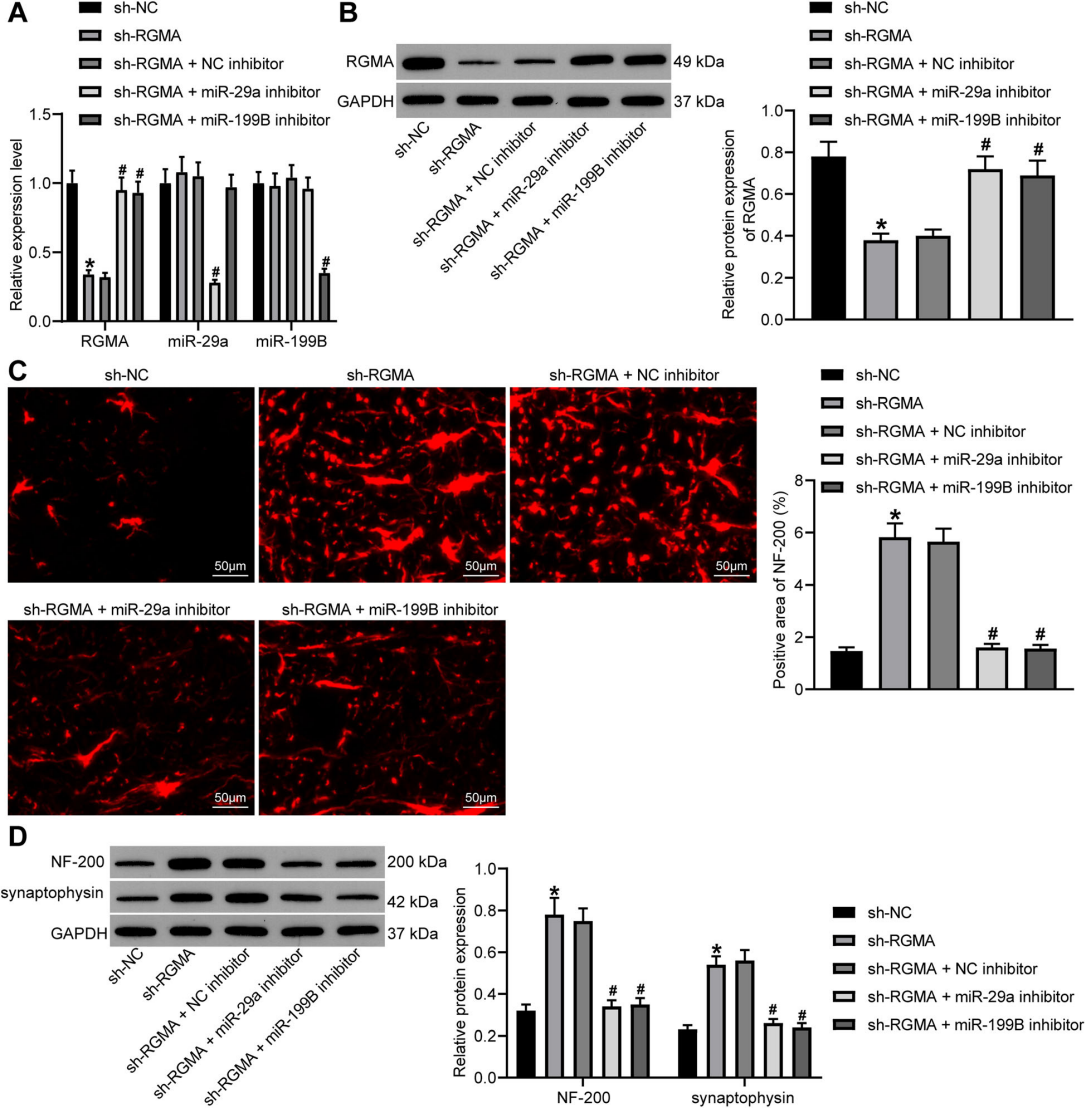
Silencing of RGMA promotes the neurological recovery in rats following SCI. **a** Expression of miR-29a, miR-199B, and mRNA of RGMA in gray matter determined by RT-qPCR. **b** Protein level of RGMA in gray matter evaluated by western blot analysis. **c** NF-200 expression in the gray matter 2–3 mm posterior to the injury sites using immunofluorescence staining. **d** Protein levels of NF-200 and synaptophysin in the gray matter 2–3 mm posterior to the injury sites detected by western blot analysis. *N* = 8. Data were presented as mean ± SD from at least three independent experiments. Data were compared by one-way or two-way ANOVA. *p < 0.05 vs sh-NC; ^#^*p* < 0.05 vs sh-RGMA + NC inhibitor

To further identify the involvements of RGMA in SCI and its interaction with miR-29a/199B, sh-RGMA and the additional miR-29a/199B inhibitor as well as the corresponding control vectors were administrated into the model rats. According to the RT-qPCR and western blot assays, it was found that the mRNA and protein expression of RGMA was reduced in the tissues in gray matter in SCI rats after sh-RGMA treatment, while the additional downregulation of miR-29a or miR-199B recovered the expression of RGMA in tissues (all *p* < 0.05) (Fig. 4a, b).

Still, the NF-200 expression in gray matter was determined by immunofluorescence staining. It was found that the staining intensity of NF-200 was notably increased upon RGMA silencing but then reduced by the additional downregulation of miR-29a or miR-199B (all *p* < 0.05) (Fig. 4c). Similar trends were reproduced in the western blot analysis concerning NF-200 and synaptophysin expression. The protein levels of NF-200 and synaptophysin in tissues were notably enhanced by sh-RGMA but then reduced by miR-29a or miR-199B inhibitor (all *p* < 0.05) (Fig. 4d).

### Silencing of RGMA suppresses STAT3 signaling pathway to promote the locomotor function recovery in SCI rats

**Fig. 5:**
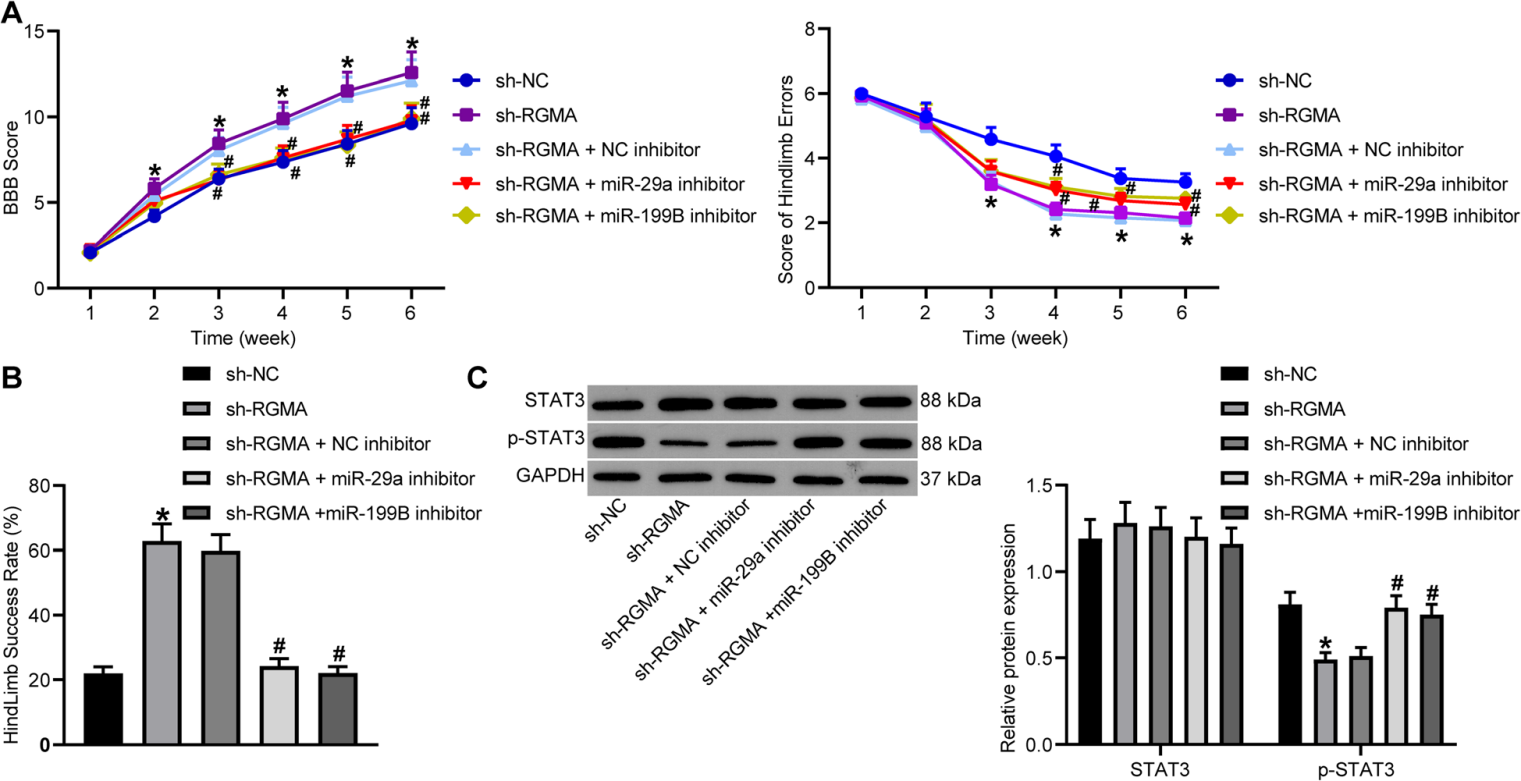
Silencing of RGMA suppresses STAT3 signaling pathway to promote the locomotor function recovery in SCI rats. **a** Weekly locomotor function of rats evaluated using the BBB rating scale. **b** Coordination of rat hindlimbs measured by a ladder-climbing test. **c** Total protein and phosphorylation of STAT3 in gray matter measured by western blot analysis. *N* = 8. Data were presented as mean ± SD from at least three independent experiments. Data were compared by one-way or two-way ANOVA. *p < 0.05 vs sh-NC; ^#^*p* < 0.05 vs sh-RGMA + NC inhibitor

As for the locomotor function in rats, we further found that the BBC locomotor scores of model rats were notably enhanced after sh-RGMA transfection from the 2nd week following SCI (*p* < 0.05), but the scores were reduced by the additional administration of miR-29a/199B inhibitor since the 3rd week (Fig. 5a). The ladder-climbing test results showed that the number of times of slips (error) was reduced since the 3rd week when RGMA was silenced, companying with an increase in the success step rate. However, the improvements in BBB locomotor scores and the coordination in rat hindlimbs were blocked by miR-29a/199B inhibitor from the 4th week (all *p* < 0.05) (Fig. 5b).

We then measured the STAT3 signaling pathway activity in gray matter. It was found that silencing of RGMA led to a significant decline in STAT3 phosphorylation, but this inhibition was then blocked by the further downregulation of miR-29a and miR-199B (all *p* < 0.05). During these processes, the total protein level of STAT3 showed little changes (*p* > 0.05) (Fig. 5c).

## Discussion

SCI has an annual incidence of 10.4–83 million cases globally and bring long-term heavy burdens to patients and the healthy management organizations [24, 25]. miRNAs have emerged as either biomarkers or therapeutic options for SCI management due to their versatile functions in key events such as oligodendrocyte development, axonal regeneration, and remyelination [7, 26]. In the present research, we validated the potent functions of miR-29a/199B in neurologic and locomotor functional recovery in a rat model with the involvement of downregulation of RGMA and the suppression of the STAT3 signaling pathway.

After identification of the successful model establishment, the initial finding of the study was that miR-29a and miR-199B were notably downregulated in the SCI model rats, which was in line with the previous studies [8, 9]. Neural network reconstruction is a key event for functional recovery after SCI. Next, we found that overexpression of miR-29a/199B in the gray matter in lesion sites led to an increase in the levels of NF-200 and synaptophysin. NF-200 is a neurofilament protein that represents a typical biomarker for mature neuronal cells in the CNS, and thus an effective marker for nerve regeneration [27], while synaptophysin usually serves as an important marker of synaptic density [28]. In addition, the BBB rating scale suggested that the locomotor score of rats was increased when miR-29a and miR-199B was upregulated. More obviously, the ladder-climbing test suggested that the coordination of rat hindlimb steps was improved upon miR-29a/199B overexpression. The promoting role of miR-29a in nerve regeneration has been well reported. It was reported to regulate axon branching by directly controlling doublecortin in primary neurons [29]. Likewise, miR-29a was found to be upregulated and was possibly responsible for Kuwanon V-induced neurogenesis [30]. miR-29a has been established to have the potency to promote neurite outgrowth in N2a cells [31]. Though miR-199B is relatively less concerned, it has been noted to be poorly expressed and suppress hypoxia-inducible factor-1 alpha to counter the progression of human intractable epilepsy [32]. Here, we validated the neurological recovery promotion of these two miRNAs.

miRNAs exert their functions primarily by binding to the diverse downstream mRNAs. Here, the integrated mRNA prediction on StarBase system and the dual-luciferase reporter gene assays validated that miR-29a and miR-199B shared a binding relationship with RGMA. Then, we found RGMA was upregulated in the gray matter in lesion sites in model rats. RGMA is a potent suppressor of axon regeneration and neurite outgrowth [33]. It was reported to express in the glial scar and myelin and is accumulated in lesioned sites after a traumatic injury of the spinal cord and brains in rodents [10]. In addition to rodents, administration of neutralizing antibody against RGMA was found to lead to recovery from impaired manual dexterity in a monkey model of SCI [12]. On this basis, we further modified RGMA expression in the SCI rat models. Consequently, artificial downregulation of RGMA led to an increase in the levels of NF-200 and synaptophysin, and the locomotor function and coordination of rats were improved. However, the promoting roles of sh-RGMA were blocked by the further downregulation of miR-29a and miR-199B, indicating that silencing of RGMA is accountable for miR-29a/199B-mediated neurologic function recovery.

Either miR-29a or miR-199B has been reported to regulate the STAT3 signaling pathway [22, 23], which is a well-known transcription factor especially participating in cell proliferation and inflammation in the lesion site after SCI, including inducing astrocyte proliferation, astrogliosis, and scar formation [15, 34]. Similarly, activation of STAT3 has been demonstrated to suppress nerve regeneration and repair and induce neuropathic pain and inflammation [21, 35, 36]. Here, our study found that phosphorylation of STAT3 was increased in the SCI model rats, and miR-29a or miR-199B upregulation was found to suppress this trend. Interestingly, silencing of RGMA was also observed to suppress the STAT3 phosphorylation, indicating that STAT3 activation is possibly implicated in RGMA-mediated nerve degeneration inhibition.

## Conclusion

To conclude, our study evidenced that miR-29a/199B can promote neurologic recovery and improve the locomotor function of rats with SCI through suppressing RGMA expression, which leads to further downregulation of STAT3. However, there remains a major limitation in this research that although we evidenced RGMA silencing suppresses STAT3 phosphorylation, the detailed regulatory network and the exact involvement of STAT3 in RGMA mediation need further evidence. We would like to perform rescue experiments to validate the interactions between RGMA and STAT3 phosphorylation and their joint effects on neuronal growth in the near future. We also hope more studies will be conducted in this field to provide more understandings in the molecular mechanism in SCI and offer more ideas for SCI control.

## Data Availability

All the data generated or analyzed during this study are included in this published article.

## Abbreviations

ANOVA: Analysis of variance
BBB: Basso, Beattie, and Bresnahan
CNS: Central nervous system
GAPDH: Glyceraldehyde-3-phosphate dehydrogenase
HRP: Horseradish peroxidase
IgG: Immunoglobulin G
LV: Lentiviral vector
mean ± SD: Mean ± standard deviation
miRNAs: MicroRNAs
MUT: Mutant type
NC: Negative control
RGMA: Repulsive guidance molecule A
RT-qPCR: Reverse transcription-quantitative polymerase chain reaction
SCI: Spinal cord injury
shRNA: Short hairpin RNA
SPF: Specific-pathogen-free
STAT3: Signal transducer and activator of transcription 3
WT: Wild-type
